# OCT evaluation of orthodontic surface sealants: a 12-month follow-up randomized clinical trial

**DOI:** 10.1007/s00784-020-03462-7

**Published:** 2020-08-13

**Authors:** Sinan Şen, Ralf Erber, Gül Orhan, Sebastian Zingler, Christopher J. Lux

**Affiliations:** Department of Orthodontics and Dentofacial Orthopaedics, University of Heidelberg, Im Neuenheimer Feld 400, 69120 Heidelberg, Germany

**Keywords:** Orthodontic treatment, Demineralization, Surface sealants, Abrasion, Tooth cleaning procedures

## Abstract

**Objectives:**

The aim of this single-center randomized controlled trial (NCT03753256) was to assess orthodontic surface sealant layer thickness and integrity in vivo during a 12-month follow-up by optical coherence tomography (OCT).

**Materials and methods:**

Using a split-mouth design, quadrants of 20 patients treated with fixed orthodontic appliances were included. Quadrants were randomly assigned to the sealants Pro Seal® (PS) or Opal® Seal™ (OS). OCT scans were performed immediately after the application of the sealants and after 3, 6, 9, and 12 months. Sealant layer thicknesses and their integrity were determined at 5 regions of interest (ROIs) known for high risks of demineralization. Sealant integrity loss was determined using a self-developed scale.

**Results:**

A total of 16 patients successfully completed the study. The studied sealants showed significant differences in initial layer thickness. Mean layer thickness was significantly lower for PS (67.8 μm, (95% CI, 56.1–79.5)) than for OS (110.7 μm, (95% CI, 97.3–124.1)). Layer thickness loss was significant after 3 months for PS and after 6 months for OS. Sealant integrity was compromised in more than 50% of the ROIs already after 3 months for both sealants.

**Conclusions:**

Patients treated with fixed orthodontic surface sealants lost the integrity of the protective layer in more than 50% of cases after 3 months, and the layer thickness of the sealants was significantly reduced after 3–6 months.

**Clinical relevance:**

The protective effect against demineralization lesions of orthodontic sealants in patients treated with fixed appliances appears to be limited in time. Further preventive measures should be investigated.

**Trial registration:**

ClinicalTrials.gov (NCT 03753256)

**Electronic supplementary material:**

The online version of this article (10.1007/s00784-020-03462-7) contains supplementary material, which is available to authorized users.

## Introduction

The application of orthodontic surface sealants became one of the most popular methods to prevent demineralization during orthodontic treatment with fixed appliances, partly also because these materials substitute conventional bracket adhesives in commonly used pre-bonding applications. In various in vitro studies, it has been shown that surface sealants, as a mechanical barrier, can protect treated surfaces against microbiological, chemical, and thermal interactions.

For instance, Premaraj et al. tested two commercially available orthodontic sealants Pro Seal® (PS) and Opal® Seal™ (OS) against acid resistance. They could show that both sealants can protect enamel surfaces from 0.1 M lactic acid (pH 4.5) penetration after 4 weeks of exposure. They also evaluated adherence of *Streptococcus mutans* and Lactobacilli on both sealants and found significantly higher *S. mutans* adherence on PS than on OS, whereas *Lactobacillu*s adherence was comparable between both sealants. Moreover, they found significantly higher levels of released fluoride (F) from PS than from OS. However, the authors doubt its clinical relevancy, because no continuous release could be observed from both sealants [1].

In an in vitro study by Coordes et al., three surface sealants (Pro Seal®, Alpha-Glaze®, and Seal&Protect®) were tested using thermal cycling (1000 cycles, 5° and 55 °C), mechanical loading (only linear cleaning movements of a soft tooth brush with a contact pressure of 1 N/cm^2^, 1000 cycles of 25 min cleaning time), and chemical loading using lactic acid (pH 4.6) for 7 days. They found that only PS protected teeth remained free from demineralization, whereas lesion depths up to 120 μm were detected in teeth protected by the other sealants [2].

The integrity of the layer is essential for the sealants to function as a mechanical barrier against early demineralization and the development of white spot lesions. After in vitro demineralization Frazier, Southard et al. could detect impaired integrity of the sealant layer in 20% of sealed and bracketed teeth. The analysis of teeth sections using polarized light microscopy revealed demineralization in areas with impaired integrity of the sealant layer. Lesion depths of such demineralization areas were similar to those observed in the control group [3].

The outcomes regarding the abrasion behavior of sealants such as LED ProSeal®, LightBond™, OrthoSolo™, Seal&Protect®, Pro Seal® (PS), and Opal® Seal™ (OS) have already been extensively investigated by different groups using in vitro experimental setups. In summary, these studies showed a striking abrasion both by simulating dental hygiene at home and professional tooth cleaning including air-powder polishing using sodium bicarbonate or glycine powder [4–6].

The evidence from in vitro studies on the integrity of surface sealants was investigated in a clinical trial. Knosel et al. assessed the durability of the surface sealant Opal® Seal™ by scoring the material layer integrity using a black light UV lamp. They found that sealant integrity was very poor already after 3.5 months, so that < 50% of the sealant or no sealant was left on the labial surface. These authors emphasized the need of further clinical trials assessing the durability of surface sealants [7].

Thus, seeking a tool to non-invasively assess surface sealant layer thickness and integrity longitudinally in patients, we recently showed that optical coherence tomography (OCT) applied as a cross-sectional imaging method can successfully be used for the assessment of orthodontic surface sealant layer abrasion in vitro and in vivo [8].

In a previous work using OCT, we showed surface sealants layer thickness reduction after professional tooth cleaning in vitro and in vivo in patients wearing fixed appliances [9]. The purpose of the present single-center randomized controlled trial was to evaluate surface sealant thickness and integrity during a 12-month follow-up in patients wearing fixed appliances using OCT.

## Materials and methods

### Study design

This single-center randomized controlled trial (RCT) adheres to the standards of the CONSORT 2010 Statement [10].

The study protocol of this present study was approved by the ethics committee of the Medical Faculty of the Heidelberg University (approval no.: S-370/2015), and the trial was registered on ClinicalTrials.gov (NCT 03753256). The study conformed to the Declaration of Helsinki and was performed in accordance with the European Medicines Agency Guidelines for Good Clinical Practice. Before participation, all patients or their parents/legal guardians received oral and written study information and signed a written consent form.

The original study protocol, which included four study arms by combining two different orthodontic sealant materials and two different tooth cleaning procedures, was recently published [9]. Since the focus of the current report is the longitudinal observation of the abrasion behavior and integrity of the two included surface sealants, within this manuscript, the study will be presented and discussed as a 2-arm RCT.

### Study population, randomization, and blinding

From May 2017 to December 2017, 20 consecutive orthodontic patients prior to treatment with fixed appliances were included in the study by interns and residents, including the authors of the present study, at the Department of Orthodontics and Dentofacial Orthopedics, Dental School, University of Heidelberg.

Quadrants of the included 20 participants were randomized by an external randomizing center. To ensure a balance in sample size across groups, block randomization was used (four quadrants in each block). As reported previously, due to the different optical properties of the sealants used in this study which makes them easily identifiable, blinding of raters was not possible [9]. Thus, neither patients nor clinical practitioners and raters were blinded.

### Interventions: application of pre-bonding orthodontic surface sealants and PTC procedures

The orthodontic surface sealants Opal® Seal™ (Opal Orthodontics, Utah, USA, Lot. No BF6D7) and Pro Seal® (Reliance Orthodontic Products, Itasca, USA, Lot. No. 175185) were used according to the manufacturers’ instructions as a primer before bonding the brackets (Discovery smart, Dentaurum, Ispringen, Germany) by two experienced orthodontists.

PTC procedures were performed as previously described [9] by two dental hygienists with over 20 years of experience on the job. As suggested by Migliorati et al. [11], PTC procedures were performed every 3 months, after the integration of the fixed appliance during the observation time of 12 months.

### Study outcomes: longitudinal assessment of sealant thickness and integrity using OCT imaging

Using a modified ophthalmic OCT device (Spectralis®; Heidelberg Engineering GmbH), thickness and integrity of sealant materials were assessed longitudinally on the central incisors. Volume scan mode was chosen to perform stacks of cross-sectional OCT images which were perpendicular to the labial tooth surface, parallel oriented to the bracket slots and with a layer width of 99 μm as previously reported [8, 9].

**Fig. 1:**
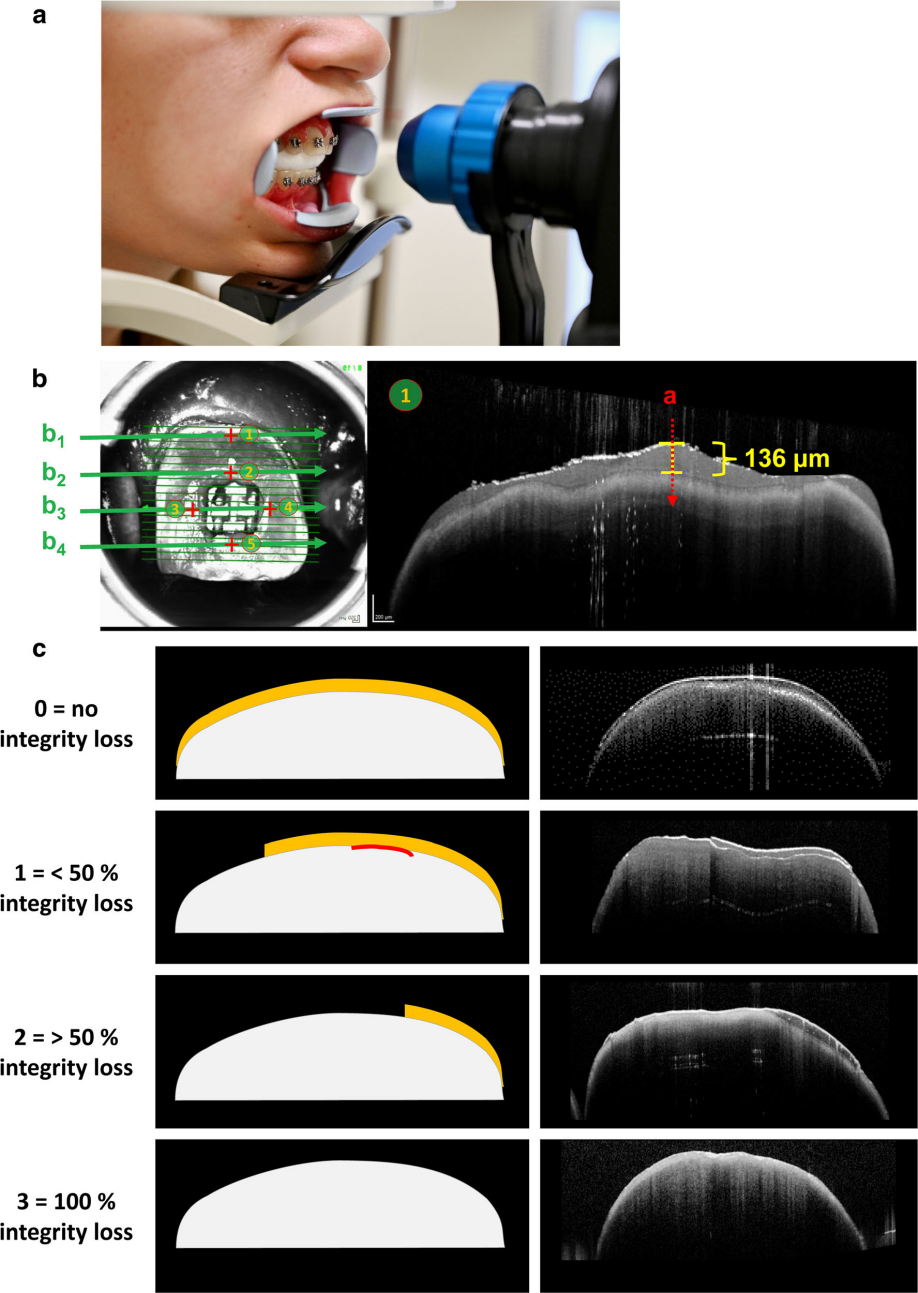
Study outcomes by OCT evaluation. **A** OCT measurements in a clinical setting: Patients rested their chin and forehead or glabella against the supporting surfaces of the device during the OCT image acquisition. A retractor for the cheek and lips was used to expose the scanning area. A cotton roll was used to uncover the teeth of the lower jaw. **B** Sealant thickness measurements on the bracket bonded central incisors. Left: 5 regions of interests (ROI) on the labial tooth surface with high risks of demineralization were examined―1 mm from the gingival margin (1) and 1 mm from the bracket in four directions, gingival (2), mesial (3), distal (4), and incisal (5). Right: Exemplified surface sealant layer thickness measurement using cross-sectional OCT images (b-scans) perpendicular to the labial tooth surface. The b-scans were oriented parallel to the bracket slots and four b-scans (b1–b4) were chosen to capture 5 ROIs. **C** A schematic representation of the criteria established for the scoring of sealant integrity loss based on cross-sectional OCT images. Sealant integrity loss on all cross sections was determined using the following scale: 0 (no integrity loss), 1 (< 50% integrity loss), 2 (> 50% integrity loss), and 3 (100% integrity loss). Left column: Illustration of the integrity loss scale. For better visualization sealants are depicted in yellow, tooth structures in white and gap formation in red. Right column: representative OCT cross-sectional images

Sealant thicknesses and their integrity are determined at 5 regions of interest (ROIs) known for high risks of demineralization [12, 13]: 1 mm from the gingival margin and 1 mm from the bracket in four directions (gingival, mesial, distal, and incisal, Fig. 1B). To assess the material thickness at each of 5 cross-sectional OCT images, the previously validated “tracking point model” was used [8, 9].

Sealant integrity loss on all investigated cross-sectional areas was determined in accordance with the Knosel et al. [7] and in analogy to the ARI index using the following classification: 0 (no integrity loss), 1 (< 50% integrity loss), 2 (> 50% integrity loss), and 3 (100% integrity loss). Criteria of the scale are represented graphically in Fig. 1C.

OCT imaging was performed, and both outcomes were analyzed by two experienced raters (S. Sen and G. Orhan).

### Sample size calculation and statistical methods

As described previously [9], sample size calculation was performed for the outcome demineralization development during orthodontic treatment. Based on the study of O’Reilly et al., we assumed that the mean difference of the incidence of white spot lesions between surface sealant treated and untreated teeth would be 31% with a standard deviation of 16.75% [14]. At a desired power of 0.99 and alpha = 0.01, the sample size calculation yielded a total of 16 patients, considering possible dropouts, we included 20 patients into our study. Surface sealant layer thickness data were analyzed using Kruskal-Wallis one-way analysis of variance and Tukey post-hoc tests. Sealant integrity loss data were analyzed using Mann-Whitney *U* test. Pairwise comparisons were performed and *p* values < 0.05 were considered as statistically significant. Data were processed, and graphs were created using SigmaPlot (version 14.0, Systat Software Inc., San Jose, CA, USA).

## Results

The main objective of this randomized clinical trial was to longitudinally investigate the surface layer thickness and the integrity of orthodontic surface sealants in patients under clinical conditions.

### Study flow

**Fig. 2:**
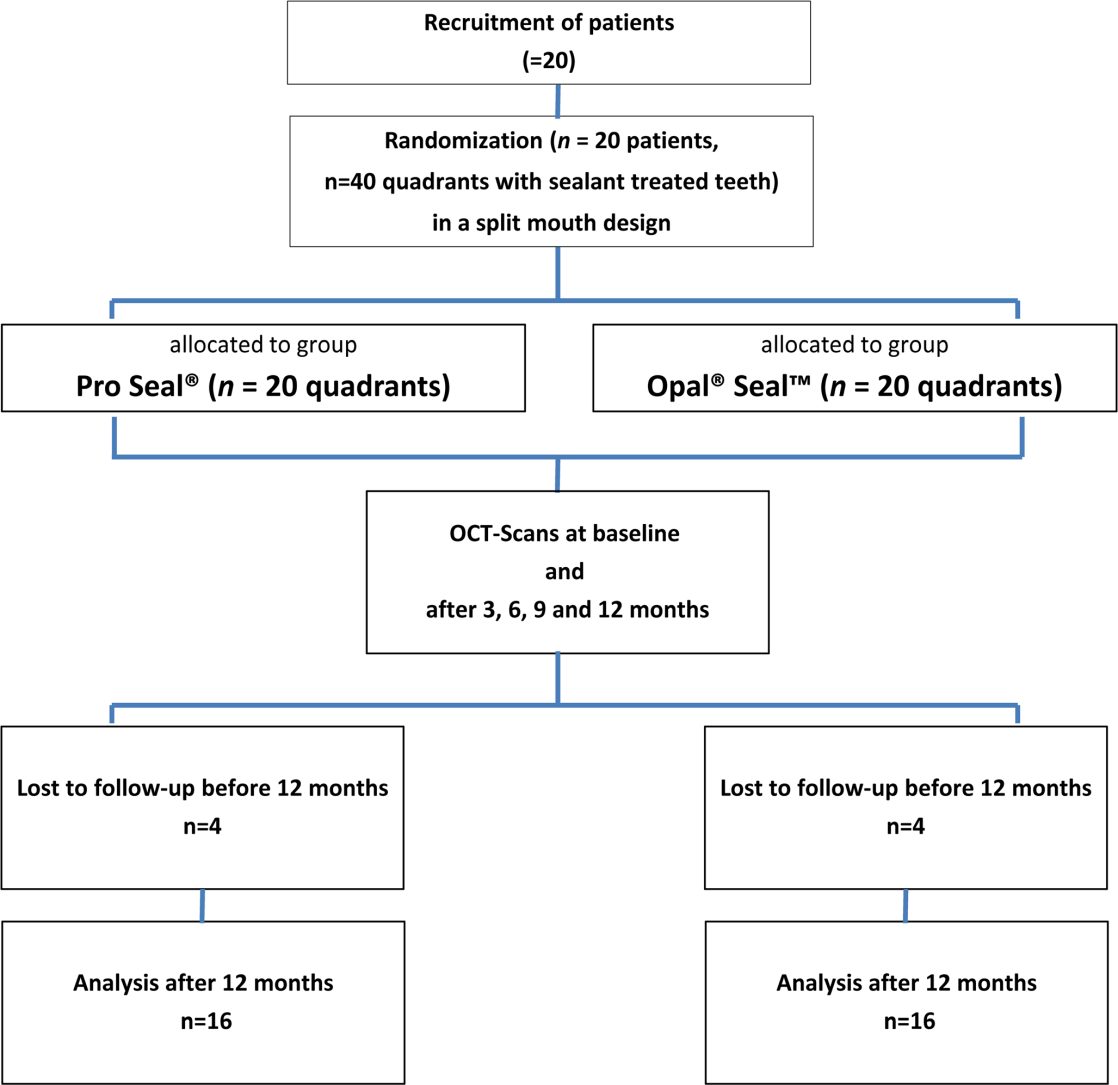
Flowcharts of patients and outcome measures

In this randomized clinical trial, split-mouth design was used to assess surface sealant layer thickness and integrity of Pro Seal® (PS) and Opal® Seal™ longitudinally for 12 months of follow-up. Sixteen of 20 initially included patients could be analyzed. Unfortunately, four patients did not attend OCT at least once and were excluded from the study. Five ROIs on a middle incisor treated with PS or OS were analyzed per sample. This resulted in a total of 80 ROIs per group and time point. No harm or unintended effects were observed during the whole study period. The study flow diagram is depicted in Fig. 2.

### Initial material thickness on different location

**Fig. 3:**
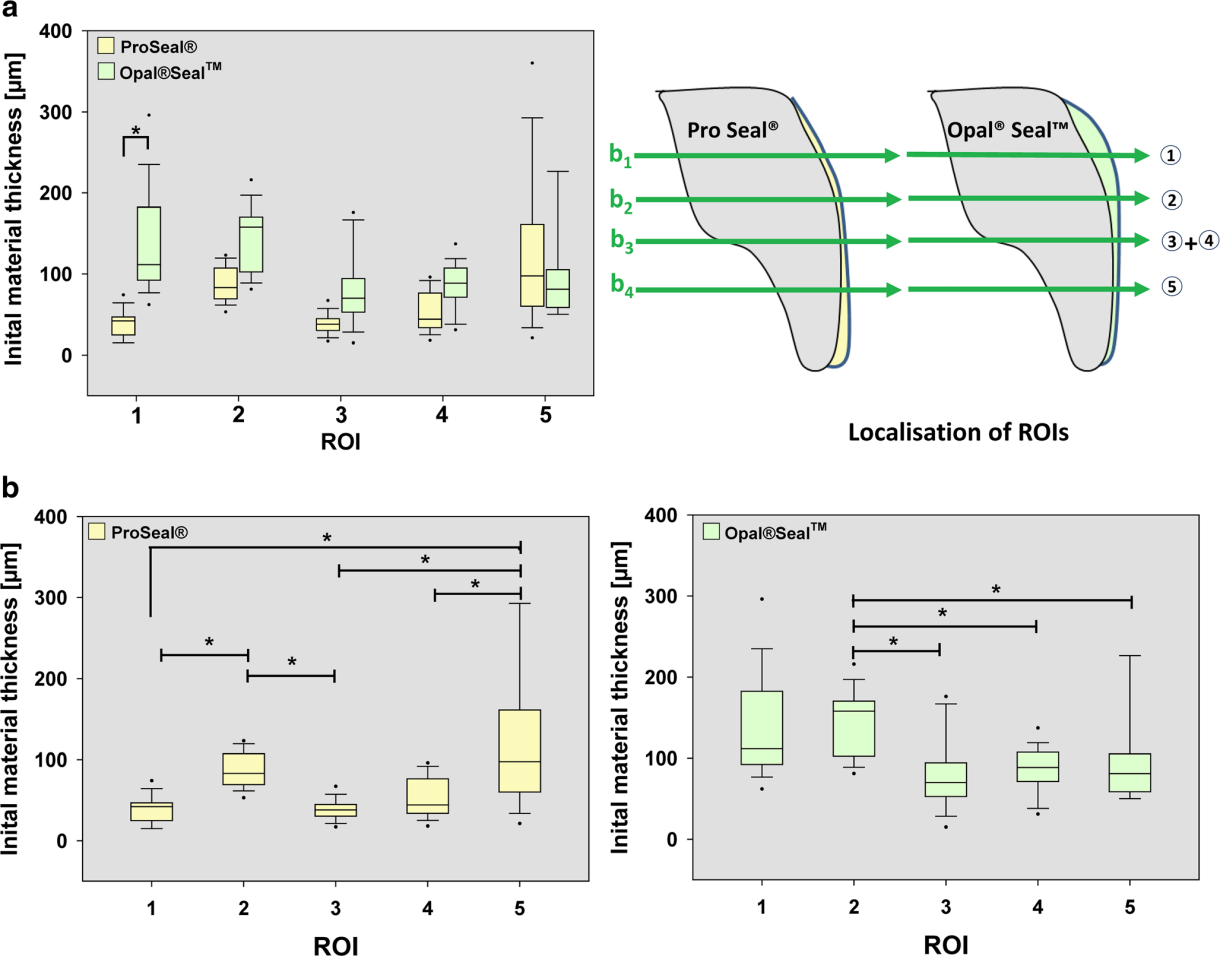
Boxplots and a schematic illustration of initial material layer thickness. **A** Box plots comparing initial sealant layer thicknesses between the sealant groups at the different ROIs. Localization of the ROIs (1–5) is schematically depicted on the right. A significant difference between PS and OS was detected at the most gingival measurement point (ROI 1), and apparent differences between the sealants at the other ROIs. **B** Box plots comparing initial sealant layer thicknesses at different ROIs () within the sealant groups. PS (left), OS (right)

The initial thickness of the sealant layer varied in the 5 regions of interests (ROIs) studied for both Pro Seal® (PS) and Opal® Seal™ (OS). Layer thickness of PS decreased from incisal to gingival ROIs, and the layer thickness measured at the most gingival measurement point (ROI 1) was significantly smaller (*p* < 0.001) than the layer thickness at the ROIs. For OS, the thickness at (ROI 2) measurement point 2 (1 mm gingival to bracket edge) was significantly greater (*p* < 0.05) than the thickness measured at the other ROIs. At the most gingival measurement point (ROI 1), both sealant materials differ significantly (*p* < 0.001) (Fig. 3). Mean layer thickness in 5 regions was significantly lower in PS-treated teeth (67.8 μm, (95% CI, 56.1–79.5)) than in OS-treated teeth (110.7 μm, (95% CI, 97.3–124.1)).

### Significant reduction of surface sealant layer thickness

Under clinical conditions, both surface sealants showed a significant reduction of mean layer thickness measured at 5 ROIs during the 12-month observation period. The layer thickness reduction of Pro Seal® (PS) was significant (*p* = 0.006) already after 3 months, whereas surface sealant layer thickness reduction of Opal® Seal™ (OS) was significant (*p* < 0.001) after 6 months.

**Fig. 4:**
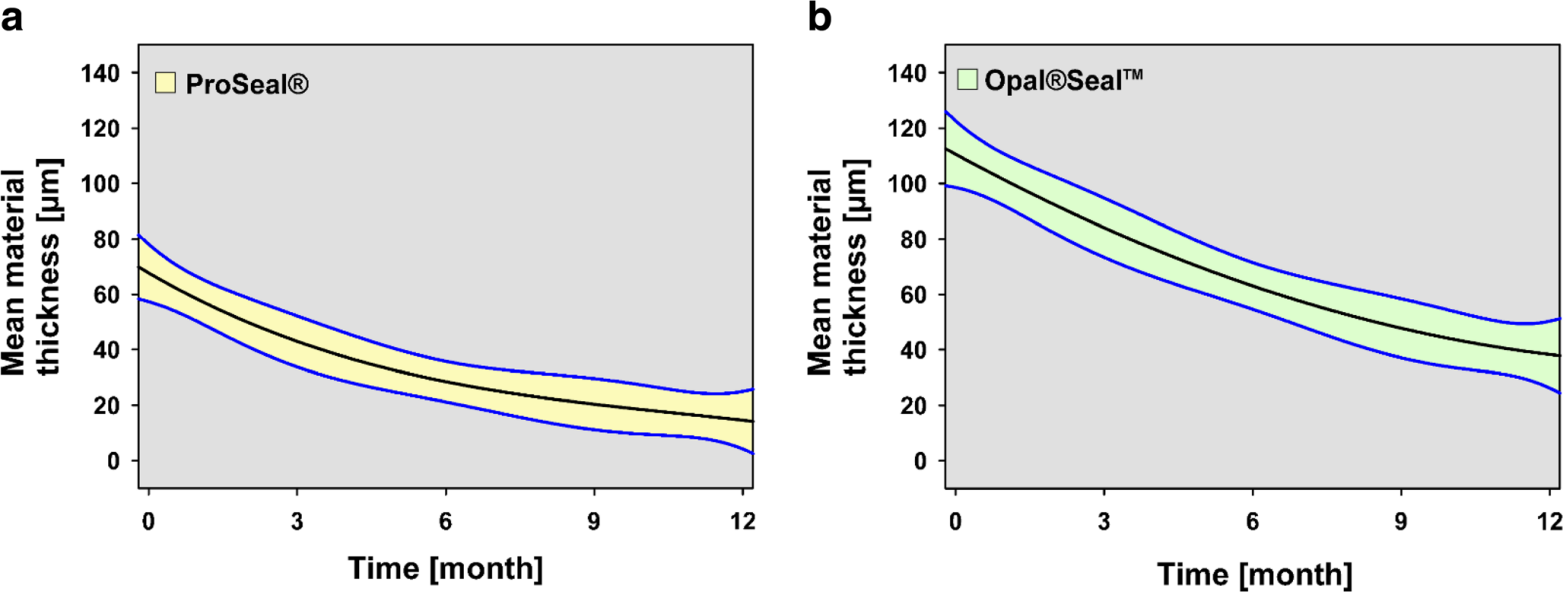
Longitudinal view of sealant layer thickness during the 12-month observation period. Layer thickness reduction of Pro Seal® (**a**) and Opal® Seal™ (**b**) during the 12-month observation period thick midlines in the graphs represent the smoothed local mean with point wise 95% confidence intervals (yellow and green areas)

**Fig. 5:**
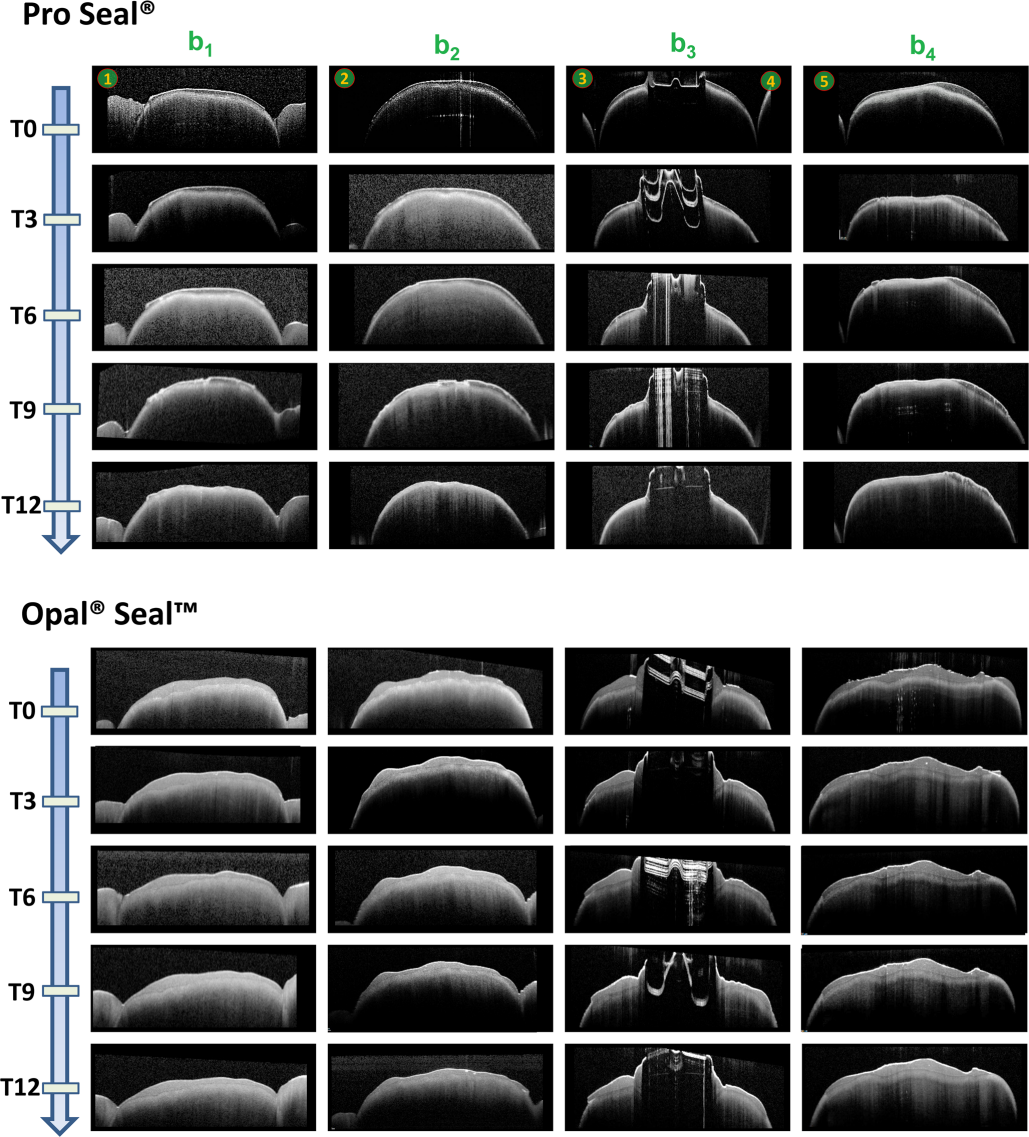
Representative OCT images for both sealants during the 12 months of observation at different ROIs. The four cross-sectional OCT scan images (b-scans, b1–b4) are oriented in parallel to the bracket slots to capture 5 ROIs (numbers in circles 1–5, see also Figs. 1B and 3A for details). Layer thickness reduction and integrity loss of both sealants show the substantial functional loss of both materials

The mean material thicknesses measured in 5 ROIs at baseline, at the control examinations 3, 6, 9, and 12 months after the integration of the fixed appliance, are presented in Table 1; a graphical representation of the data is provided in Fig. 4; and representative OCT images are shown in Fig. 5.

**Table 1:** Mean sealant layer thickness during the 12-month observation period

Material	Time point	Mean layer thickness (95% CI) (μm)	*p* value (vs. baseline)
Pro Seal®			
	Baseline	67.81 (56.11–79.51)	-
	3 m	43.16 (31.92–54.40)	*0.006
	6 m	28.46 (17.83–39.10)	*< 0.001
	9 m	20.61 (10.56–30.67)	*< 0.001
	12 m	14.71 (5.48–23.94)	*< 0.001
Opal®Seal™			
	Baseline	110.69 (97.28–124.10)	–
	3 m	83.95 (71.47–96.43)	0.495
	6 m	63.39 (51.14–75.64)	*< 0.001
	9 m	47.65 (35.64–59.66)	*< 0.001
	12 m	38.38 (27.10–49.65)	*< 0.001

*n* = 80 ROIs per group and per time point, 95% CI = 95% confidence interval, *p < 0.05 (ANOVA, Tukey post-hoc test)

In the oral cavity, time-dependent decreases of mean layer thicknesses were observed for PS and OS. Slop estimates and 95% CIs are presented in Table 2. The rates of material thickness loss were calculated in μm per month. These were significantly higher for OS (approx. 6 μm per month) compared with PS (approx. 4.3 μm per month).

**Table 2:** Calculated slopes of material thickness loss per month under clinical conditions

Material	Slope (95% CI) (μm/month)	*p* value (PS vs. OS)
Pro Seal®	− 4.2917 (− 4.8551, − 3.7283)	*0.044
Opal®Seal™	− 6.0308 (− 6.6825, − 5.3791)	

*n* = 80, 95% CI = 95% confidence interval, *p < 0.05 (ANOVA, Tukey post-hoc test)

### Substantial loss of integrity of both sealants for 12-month follow-up

The extent of sealant defects at enamel interfaces was assessed every 3 months during the 12-month observation period using OCT-generated optical cross sections in 4 sectional planes (see Fig. 3A for their exact location). For this purpose, linear measurements of the distances covered with sealer were carried out and put in relation to the total distance recorded in the optical cross-section. The integrity loss was categorized according to the study by Knosel and colleagues [7]: (no integrity loss), 1 (< 50% integrity loss), 2 (> 50% integrity loss), and 3 (100% integrity loss).

**Fig. 6:**
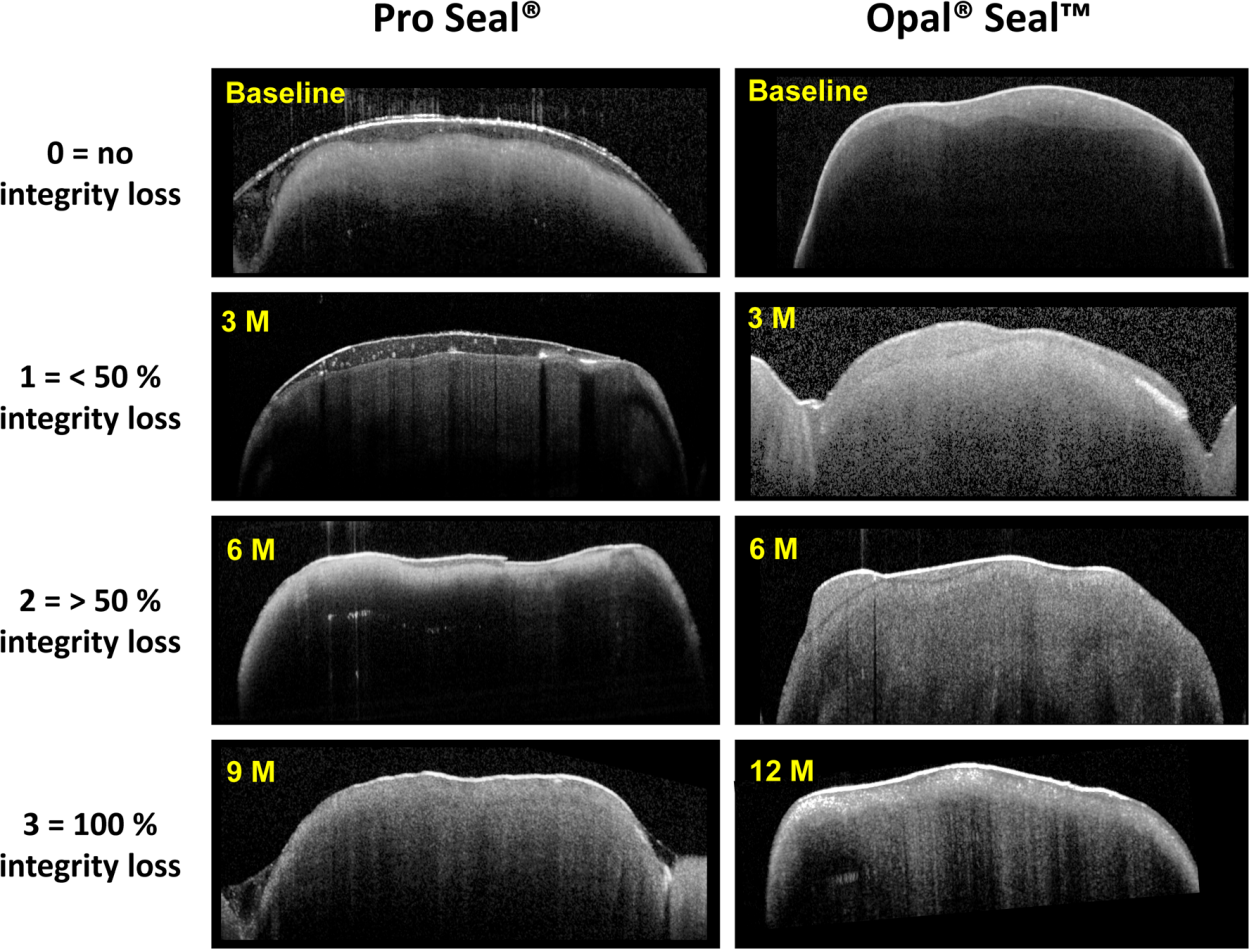
Representative OCT images for both sealants showing different levels of impaired integrity (score 0–3) including up to 100% loss of surface sealant

Already after 3 months, both sealants revealed impaired integrity (category 1–3) of the surface layer in more than 50% of the sealant-treated tooth surfaces (Table 3). Significant differences regarding integrity loss were evident between the sealants after 3, 9, and 12 months (*p* < 0.05). Representative OCT images are shown in Fig. 6.

**Table 3:** Scores for sealant integrity loss at 3, 6, 9 and 12 months

Material	Observation time (months)				
	3 m		6 m	9 m	12 m
$$ \frac{\begin{array}{l}\mathrm{Pro}\ \mathrm{Seal}\circledR \\ {}\mathrm{percentage}\ \mathrm{of}\ \mathrm{individual}\ \mathrm{scores}\end{array}}{\mathrm{percentage}\ \mathrm{of}\ \mathrm{scores}>0} $$	%	$$ \frac{0\kern1.00em 1\kern1.00em 2\kern1.00em 3}{\frac{\begin{array}{cccc}15& 58.75& 18.75& 7.5\end{array}}{\begin{array}{cccc}& 85\ast & & \end{array}}} $$	$$ {\displaystyle \begin{array}{l}\frac{\begin{array}{cccc}0& & 1& \end{array}\kern0.5em 2\kern1.00em 3}{\frac{\begin{array}{cccc}6.25& 45& 25& 23.75\end{array}}{\begin{array}{cccc}& 93.75& & \end{array}}}\\ {}\end{array}} $$	$$ {\displaystyle \begin{array}{l}\frac{\begin{array}{cccc}0& & 1& \end{array}\kern0.5em 2\kern1.00em 3}{\frac{\begin{array}{cccc}1.25& 38.75& 33.75& 26.25\end{array}}{\begin{array}{cccc}& 98.75\ast & & \end{array}}}\\ {}\end{array}} $$	$$ {\displaystyle \begin{array}{l}\frac{\begin{array}{cccc}0& & 1& \end{array}\kern0.5em 2\kern1.00em 3}{\frac{\begin{array}{cccc}0& 31.25& 27.5& 41.25\end{array}}{\begin{array}{cccc}& 100\ast & & \end{array}}}\\ {}\end{array}} $$
$$ \frac{\begin{array}{l}\mathrm{Opal}\circledR \mathrm{Seal}\texttrademark \\ {}\mathrm{percentage}\ \mathrm{of}\ \mathrm{individual}\ \mathrm{scores}\end{array}}{\mathrm{percentageofscores}>0} $$	%	$$ \frac{\begin{array}{cccc}0& & 1& \end{array}\kern0.5em 2\kern1.00em 3}{\frac{\begin{array}{cccc}38& 52.5& 8.75& 1.25\end{array}}{62.5\ast }} $$	$$ \frac{\begin{array}{cccc}0& & 1& \end{array}\kern0.5em 2\kern1.00em 3}{\frac{\begin{array}{cccc}16.25& 63.75& 16.25& 3.75\end{array}}{\begin{array}{ccccc}& & 83.75& & \end{array}}} $$	$$ \frac{\begin{array}{cccc}0& & 1& \end{array}\kern0.5em 2\kern1.00em 3}{\frac{\begin{array}{cccc}11.25& 47.5& 30& 11.25\end{array}}{88.75\ast }} $$	$$ \frac{\begin{array}{cccc}0& & 1& \end{array}\kern0.5em 2\kern1.00em 3}{\frac{\begin{array}{cccc}10& 38.75& 33.75& 17.5\end{array}}{90\ast }} $$

*n* = 80, *p < 0.05 Pro Seal® vs. Opal®SealTM (Mann-Whitney *U* test)

## Discussion

To prevent demineralization during orthodontic treatment with fixed appliances, the application of orthodontic surface sealants became one of the most popular methods. These materials substitute conventional pre-bonding bracket adhesives. In various in vitro studies, it has been shown that surface sealants can act as a mechanical barrier and protect the enamel against demineralization due to microbiological, chemical, and thermal interactions. However, in contrast to the in vitro studies, according to current in vivo studies, there is no robust evidence for the prevention of demineralization lesion by orthodontic surface sealants [15, 16].

For example, O’Reilly et al. from their prospective clinical trial concluded that the clinical use of orthodontic sealants showed measurable but only negligible clinical impact on preventing white spot lesions [14]. In another randomized clinical trial, it was concluded that only excellent or at least good oral hygiene did prevent white spot lesions, whereas no significant effect on lesion incidence was observed for the surface sealant SeLECT Defence™ [17].

Also, the results obtained by the Leizer and colleagues showed no protective effect of surface sealants against decalcification. These authors compared, in a randomized clinical trial using a split-mouth design, the occurrence of decalcification on teeth protected by the fluoride-releasing filled sealant Pro Seal® (PS) with those of the unfilled non-fluoride bracket adhesive Transbond MIP. To this end, twelve blinded orthodontic professionals evaluated the outcome by scoring the demineralization based on the photographs of 177 teeth from 18 patients before and after fixed appliance treatment for 12–18 months. The clinical application of PS did not provide any additional significant protection against demineralization formation compared with unfilled non-fluoride bracket adhesive Transbond MIP. After debonding, 69% of the teeth treated with PS showed progressive demineralization, so the sealant group was close to the control group, in which the lesion prevalence was 72% [18].

Knosel et al. assessed the durability of the surface sealant Opal® Seal™ by assessing the material layer integrity by exploiting the fluorescent properties of the sealer using a black light UV lamp. They found that sealant integrity was impaired already after 3.5 months so that < 50% of the sealant or no sealant was left on the labial surfaces. The authors pointed out the need for further clinical trials investigating the surface sealant durability in vivo [7].

Surface sealants are intended to function as a mechanical barrier. Thus, the integrity of orthodontic surface sealants is indispensable for caries prevention during orthodontic treatment with appliances. In order to clarify why previous clinical studies have not provided clear evidence of a protective function against demineralization by such surface sealants, the sealant layer must be examined longitudinally in detail. Therefore, the availability of a non-invasive technique allowing for the detailed clinical longitudinal assessment of orthodontic surface sealants is a prerequisite to clarify the reasons for the disappointing clinical results of such sealants.

To achieve this, we have previously demonstrated the ability of OCT imaging to assess surface sealants in vitro and in vivo [8, 9]. Using OCT, the aim of the present single-center randomized controlled trial was to investigate the abrasion and integrity loss of two popular surface sealant on central incisors during 12 months of follow-up observation at five representative ROIs with high risks of demineralization: one ROI close to gingival margin and four ROIs at four different directions around the bracket.

Firstly, we found that under clinical conditions, sealant layer thickness varied between the sealants and the individual ROIs, although the application of the sealant was standardized. The patients were positioned on the chair parallel to the floor level, and the application direction was always from the gingiva to the incisal edge. PS showed significantly lower layer thickness at the most gingival ROI (1 mm away from the margin) compared with the layer thickness at the other ROIs and layer thickness decreased from incisal to gingival ROIs. In contrast, OS showed highest layer thickness 1 mm gingival to the bracket edge. A possible explanation for this remarkable variability regarding layer thicknesses between the two sealants is that the filler particle contents, composition and, consequently, the corresponding viscosity and flow differ. PS with a filler content of 18% appears to be more flowable than OS which contains 38% filler [6].

Furthermore, we found a significant reduction of mean layer thickness for PS after 3 months and for OS after 6 months. Previously, we have investigated surface sealant abrasion from professional tooth cleaning (PTC) procedures by assessing the sealant thicknesses before and after the initial PTC and after 3 months of active treatment with fixed appliances [9].

Obviously the abrasion by PTC also contributes to the overall result obtained in this study; however, the prolonged follow-up clearly enhances the effects of daily dental care at home and the stress caused by thermal, mechanical, and chemical loads in the oral cavity [5, 8].

In addition, by assessing for the first time sealant integrity using OCT, we show that after only 3 months, a high proportion of sealed teeth show significant, clinically relevant losses of integrity of the sealing layer.

Although OS showed a significantly slower loss of layer thickness compared with PS and, based on the data on layer thickness, clinically relevant damage would only have been expected after 6 months, the data on integrity loss indicate a loss of protective effect for OS also after only 3 months.

Since the durability surface sealants may be shorter than the treatment period with fixed equipment, reapplication of sealants at short intervals would be a conceivable treatment alternative. In fact, Knosel and colleagues [7] in their work on the durability of Opal Seal suggested reapplication after 3.5 months.

However, it is unclear how such a reapplication can be performed. The application of most surface sealants, including those used in this study, requires preconditioning by etching the enamel surface. As a reapplication is not recommended by the manufacturers, suitable application recommendations are lacking. Thus, it remains unclear whether a reapplication is possible on an existing, partially damaged sealant surface and how durable a reapplied sealant would be. Therefore, the success of a reapplication cannot be assessed to date, and there is a clear need for further clinical studies.

The data obtained in our clinical trial on the integrity of Opal® Seal™ (OS) are in accordance with the data obtained in the clinical study by Knosel and colleagues [7]. Research by Tufekci et al. on the efficacy of OS in reducing demineralization around brackets showed no significant difference between the efficacy of OS and the sole use of a conventional pre-bonding primer (Transbond XT; 3 M Unitek) [19].This also applies to the lack of effectiveness of Pro Seal® (PS) against demineralization observed in another clinical study [18]. Thus, both results might be explained by our findings of high abrasion and rapid loss of integrity of PS and OS.

In summary, we investigated for the first time the clinically important abrasion of orthodontic sealants and assessed their integrity in the oral cavity in detail by sectional imaging using OCT. In particular, cross-sectional OCT imaging showed that the clinically relevant loss of integrity of the sealant layer occurs earlier than a significant reduction of the sealant layer thickness.

Similar benefits of OCT for dental clinical imaging were also reported for composite dental materials used labial tooth surfaces by Schneider and colleagues [20], who evaluated marginal adaptations of class III/IV composite restorations in vitro and in vivo by using OCT. Haak and colleagues used OCT to investigate the outcomes of universal adhesives on non-carious cervical lesions in a randomized clinical trial with a follow-up of 12 months [21].

Although OCT is increasingly used successfully in dentistry, the methodology has still some limitations. Using a modified ophthalmologic OCT device enabled us only to perform OCT imaging on the labial and buccal surface of incisors, canines, and premolars. Thus, for an application in the oral cavity, which would also make it possible to examine molars, small handheld devices would be necessary, which are however already being developed on an experimental scale [22].

Another potential limitation of this study is that the outcomes were limited by the evaluation of four cross-sectional OCT images for each time point only on central incisors. This was, on the one hand, due to the OCT technology, which is not intra-orally applicable and on the other hand due to the not yet automated and therefore very time-consuming manual image analysis to determine the layer thickness and integrity of the sealants. In general, for the future examination of dental and oral structures not only hardware adaptation but also software solutions are necessary to enable valid and automatic evaluations in clinical routine. Meller and Schott [23] in an in vitro study and Knosel and colleagues [7] in an in vivo study used the light emissions of surface sealants after excitation with UV light to determine their integrity. Such a strategy allows a rough assessment of the surface with simple, inexpensive means and is therefore ideal for the rapid assessment of the integrity of surface sealant chairside. However, the method is not suitable for the quantitative determination of the thickness of surface sealants and has not yet been standardized for the assessment of integrity. Despite the obvious disadvantages of OCT, this complex technology was necessary for this study, since it is the only method that has so far enabled reliable, standardized thickness and integrity measurements of surface sealants in vivo.

We recently reported in vitro data indicating that plaque disclosing solutions might cause cumulative esthetic deficits in surface sealant-treated teeth [24]. Therefore, although personal dental hygiene plays a role in the stability of surface sealants, we omitted assessing dental hygiene to avoid esthetic deficits caused by the use of plaque disclosing solutions.

Based on the results of the present study indicating an early loss of protection from surface sealants against demineralization, we recommend more frequent PTC appointments (e.g., every 3 as suggested by Migliorati et al. [11]) even for patients whose teeth were treated with surface sealants, to avoid both plaque accumulation and discoloration [9].

In summary, we were able to show, by means of the clinical use of cross-sectional OCT imaging, that clinically relevant sealant damages developed in popular surface sealants already 3 months after application. Thereby conforming the results of other clinical studies [7, 11, 12, 17–19, 25], we believe that the protective effect against demineralization by orthodontic surface sealants may be limited in time, and further preventive measures should be investigated.

## Conclusion

In this clinical trial, the longitudinal layer thickness decrease and the integrity loss of the orthodontic surface sealants were investigated for the first time in patients undergoing a non-ionizing, cross-sectional imaging modality by OCT. Based on this evaluation of the sealants during the 12-month observation period, the following conclusions can be drawn:

- Initial surface sealant layer thickness can vary depending on filler rates and viscosity. 18% filled Pro Seal® yielded thinner layer thicknesses than 38% filled Opal® Seal™, especially close to the gingival margin.
- Pro Seal® showed significantly reduced surface layer thickness after 3 months and Opal® Seal™ after 6 months.
- The loss of sealant integrity for Pro Seal® and Opal® Seal™ was clinically relevant: integrity loss occurred in more than 50% of cases in areas with high risks of demineralization after only 3 months.

## Electronic supplementary material

ESM 1 (DOC 219 kb)
